# Effects of dl‐3‐n‐butylphthalide on serum lipoprotein‐associated phospholipase A2 and hypersensitive C‐reactive protein levels in acute cerebral infarction

**DOI:** 10.1002/brb3.1469

**Published:** 2019-11-13

**Authors:** Xiao‐Lei Zhang, Yin‐Tao Dong, Yi Liu, Yi Zhang, Ting‐Ting Li, Feng‐Yun Hu

**Affiliations:** ^1^ Department of Neurology Shanxi People's Hospital Taiyuan China

**Keywords:** acute cerebral infarction, butylphthalide, hypersensitive C‐reactive protein, lipoprotein‐associated phospholipase A2

## Abstract

**Objective:**

This study aims to explore the curative effect of dl‐3‐n‐butylphthalide (NBP) on patients with acute cerebral infarction (ACI) and its effects on serum lipoprotein‐associated phospholipase A2 (Lp‐PLA2) and hypersensitive C‐reactive protein (hs‐CRP) levels.

**Methods:**

A total of 136 ACI patients treated in our hospital, who met the criteria, were selected and randomly divided into two groups: control group (*n* = 60, including 28 males and 32 females) and treatment group (*n* = 76, including 32 males and 44 females). Patients in the control group were treated with routine drug therapy, while patients in the treatment group were treated with NBP on this basis. A dose of 100 ml was administered by intravenous injection for 2 times/day, for 14 days. The curative effect was evaluated using the National Institute of Health Stroke Scale (NIHSS) and Barthel index (BI) self‐care ability. The levels of the two factors in serum were measured using enzyme‐linked immunosorbent assay, and the changes in levels of these two factors in serum at different time points before and after treatment were compared between the two groups.

**Results:**

(a) Lp‐PLA2 and hs‐CRP levels in the treatment group after treatment were significantly lower than those before treatment and those in the control group after treatment (*p* < .05). (b) The NIHSS and BI scores in the treatment group were significantly lower after treatment than before treatment and those in the control group after treatment (*p* < .05).

**Conclusion:**

Dl‐3‐n‐butylphthalide can improve the expression of Lp‐PLA2 and hs‐CRP in serum in ACI patients. Furthermore, NBP has significant efficacy in inhibiting inflammation and improving neurological symptoms.

## Graphical abstract

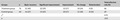

## 1 INTRODUCTION

China is a country with a high incidence of cerebrovascular disease (Chen & Wang, 2016). In China, the incidence of ischemic cerebrovascular disease accounts for 70%–80% of cerebrovascular diseases, and the mortality and disability rates of cerebral infarction are relatively high (Writing Group Members et al., 2016). Furthermore, the mortality and disability rates of cerebral infarction are also relatively high (Writing Group Members et al., 2016). Hence, the early assessment of disease severity and its timely targeted treatment have become the focus of study on cerebral infarction (Chen & Wang, 2016; Kidwell, Liebeskind, Starkman, & Saver, 2001). Dl‐3‐n‐butylphthalide (NBP) is a new class of nationally approved drug in China (Zhao et al., 2017; Tang et al., 2017), which has been widely used in clinic. Lipoprotein‐associated phospholipase A2 (Lp‐PLA2) is a new inflammatory marker (Wang, Hu, et al., 2018; Zhou, Liu, Shi, Huang, & Zhou, 2018), and its application in the treatment of atherosclerotic cardiovascular and cerebrovascular diseases has attracted much attention in recent years. Hypersensitive C‐reactive protein (hs‐CRP) is an independent risk factor for stroke that has been studied for a long period of time (Li et al., 2016). However, there are few reports on the evaluation of the condition of cerebral infarction through the combined detection of these two. The present study focused on analyzing the effects of NBP on these two indexes, Lp‐PLA2 and hs‐CRP, in order to investigate the value of NBP in the treatment of cerebral infarction.

## 2 MATERIALS AND METHODS

### 2.1 Clinical data

Double‐blind method was used in the study. All 136 acute cerebral infarction (ACI) patients, who were admitted to the Department of Neurology, Shanxi People's Hospital from May 2013 to May 2016, were enrolled into the study. On the basis of sample size formula suggested for randomized clinical trials, considering the type I error of 5% (*α* = 0.05), type II error of 20% (*β* = 0.20; power = 80%), and serum Lp‐PLA2 and hs‐CRP levels as key variables, we reached the sample size: control group (*n* = 60, 28 males and 32 females), and treatment group (*n* = 76, 32 males and 44 females), using a random number table method. The age of these patients ranged within 50–79 years old, with an average age of 64 ± 6 years old. The treatment group comprised of 76 patients, in which 32 patients were male and 44 patients were female. The age of these patients ranged within 54–81 years old, with an average age of 63 ± 6 years old. Routine blood and urine tests, biochemistry, and blood coagulation series were performed. Patients with cerebral hemorrhage or mixed hemorrhage with infarction and other encephalopathies were excluded from these two groups. This study was approved by the Ethics Committee of Shanxi People's Hospital.

### 2.2 Diagnostic criteria

The diagnosis was based on the Chinese Medical Association's diagnostic criteria for stroke and the definition of stroke formulated by the World Health Organization (WHO): the presence of focal or total ischemic lesions of the brain, and the occurrence of neurological deficits that last for more than 24 hr. The differences in age, course of disease, blood pressure, blood glucose, blood fat, size and location of infarction lesions on the computed tomography (CT) or magnetic resonance imaging (MRI) images, as well as the clinical manifestations between these two groups, were not statistically significant.

### 2.3 Treatment methods

1. Patients in the control group received conventional treatment: 100 mg/day of oral aspirin, 200 mg/day of oral atorvastatin, 0.75 mg/day of intravenous drip of citicoline, and 6 ml/day of intravenous drip of Shuxuetong injection. On the basis of the treatment in the control group, patients in the treatment group received 100 ml of intravenous drip of NBP injection, twice a day, which lasted for 2 weeks.
2. Detection of Lp‐PLA2 and hs‐CRP: Blood samples were collected from the elbow veins under the fasting state before treatment and at 14 days after treatment, and stored in a refrigerator at −80°C for detection. Lp‐PLA2 and hs‐CRP levels were detected by double‐antibody sandwich enzyme‐linked immunosorbent assay (ELISA).

### 2.4 Observation index and curative effect assessment

Neurological deficits were evaluated using the National Institutes of Health Stroke Scale (NIHSS), while the activities of daily living were assessed using the Barthel index (BI). Then, changes in the NIHSS score and BI before and after treatment were compared between these two groups. Efficacy criteria: (a) basic recovery: the neurological deficit score decreased by 91%–100%, and the disability level was 0; (b) significant improvement: the neurological deficit score decreased by 46%–90%, and the disability level was 1–3; (c) improvement: the neurological deficit score decreased by 18%–45%; (d) no change: the neurological deficit score decreased or increased by <17%; (e) deterioration: the neurological deficit score increased by >18%. Total effective rate = Basic recovery + significant improvement + improvement. The levels of specific inflammatory factors before and after treatment were observed, the serum levels of Lp‐PLA2 and hs‐CRP were measured before and after treatment, and changes in the above indicators were compared between the two groups.

### 2.5 Statistical methods

To ensure the normal distribution of variables, histogram and Kolmogorov–Smirnov test were applied. Log transformation was used for non‐normally distributed variables. We used independent samples Student's *t* test to identify between‐group differences. To determine the effects of serum Lp‐PLA2 and hs‐CRP, we applied repeated measures analysis of variance. Data were analyzed using the statistical software SPSS 19.0. Measurement data were expressed as mean ± standard deviation (*X* ± *SD*), and compared using *t* test. Count data were expressed in percentage (%), and compared using *X* ^2^ test. *p* < .05 was considered statistically significant.

## 3 RESULTS

### 3.1 Comparison of clinical efficacy between the two groups

The total effective rate of the treatment group (86%) was significantly higher than that of the control group (69%), and the difference was statistically significant (*p* < .05; Table 1). The effect in the treatment group was better than that in the control group.

**Table 1:** Comparison of clinical efficacy between two groups of cerebral infarction [*n* (%)]

Groups	*n*	Basic recovery	Significant improvement	Improvement	No change	Deterioration	Total effective rate (%)
Treatment group	76	31 (40)	22 (29)	13 (17)	10 (14)	0 (0)	86
Control group	60	16 (27)	15 (25)	10 (17)	11 (18)	8 (13)	69
*χ* ^2^							6.01
*p*							.013

### 3.2 Changes in NIHSS score and BI before and after treatment

Before treatment, the NIHSS scores and BI in treatment group (28.5 ± 5.2 and 38.8 ± 5.7) and control group (28.4 ± 5.1 and 38.9 ± 5.7) were similar, and the differences were not statistically significant (*p* > .05; Table 2). After treatment, the NIHSS score was lower in the treatment group than in the control group, while the BI was higher in the treatment group than in the control group, and the differences were statistically significant.

**Table 2:** NIHSS score and the BI rating of two groups of cases before and after treatment (*x* ± *s*)

Groups	*n*	BI		NIHSS	
		Before treatment	After treatment	Before treatment	After treatment
Treatment group	76	38.8 ± 5.7	90.2 ± 6.6	28.5 ± 5.2	7.2 ± 2.2
Control group	60	38.9 ± 5.7	72.1 ± 7.1	28.4 ± 5.1	12.5 ± 2.5
*t*		0.07	11.77	0.88	9.17
*p*		>.05	<.01	>.05	<.01

Abbreviations: BI, Barthel index; NIHSS, National Institute of Health Stroke Scale.

### 3.3 The changes in serum levels of hs‐CRP and Lp‐PLA2 in the two groups before and after treatment

Before treatment, the serum levels of hs‐CRP and Lp‐PLA2 in treatment group (11.7 ± 5.7 mg/L and 64.6 ± 7.9 ng/ml) and control group (11.6 ± 5.8 mg/L and 64.2 ± 7.9 ng/ml) were similar, and the difference was not statistically significant (*p* > .05; Table 3). After treatment, the serum levels of hs‐CRP and Lp‐PLA2 were lower in the treatment group than in the control group, and the difference was statistically significant.

**Table 3:** Case in the two groups before and after the treatment of allergic CRP and Lp‐PLA2 levels

Groups	*n*	Hs‐CRP (mg/L)		Lp‐PLA2 (ng/ml)	
		Before treatment	After treatment	Before treatment	After treatment
Treatment group	76	11.7 ± 5.7	3.5 ± 1.5	64.6 ± 7.9	33.3 ± 5.3
Control group	60	11.6 ± 5.8	5.6 ± 2.6	64.2 ± 7.9	37.5 ± 5.5
*t*		0.08	3.44	0.41	2.56
*p*		>.05	<.01	>.05	<.05

Abbreviations: hs‐CRP, hypersensitive C‐reactive protein; Lp‐PLA2, lipoprotein‐associated phospholipase A2.

## 4 DISCUSSION

Lipoprotein‐associated phospholipase A2 is a non‐Ca^2+^‐dependent serine esterase. It consists of 441 amino acids, has a relative molecular mass of 45 KD, and has a function of promoting the hydrolysis of the ester bond at the glyceryl phospholipid sn‐2 site and the degradation of platelet‐activating factors. Therefore, it is also known as platelet‐activating factor acetylhydrolase (PAF‐AH) (Dennis, Cao, Hsu, Magrioti, & Kokotos, 2011; Zhou et al., 2018). In recent years, its application in atherosclerosis‐associated cardiovascular and cerebrovascular diseases has attracted extensive attention (Alkuraishy, Al‐Gareeb, & Waheed, 2018; Esenwa & Elkind, 2016; Gorelick, 2008). Many early studies have considered that (Elkind, Tai, Coates, Paik, & Sacco, 2006; Lp‐PLA(2) Studies Collaboration et al., 2010; Oei et al., 2005) Lp‐PLA2 can hydrolyze platelet‐activating factors, inhibit thrombosis and alleviate inflammation, and work against atherosclerosis. At present, it has been considered that (Alkuraishy et al., 2018; Yang et al., 2010) some kinds of Lp‐PLA2 that can bind with high density lipoprotein (HDL) can hydrolyze oxidized phospholipids in blood, reduce the accumulation of inflammatory mediators in phagocytes, and inhibit foam cell formation, thereby exerting anti‐inflammatory and anti‐atherosclerotic effects. In the process of the hydrolysis of oxidized low‐density lipoprotein (ox‐LDL), sLp‐PLA2 produces oxidized free fatty acids (ox‐FFA) and lysolecithin (lyso‐PC) (Bonnefont‐Rousselot, 2016; Ulrich et al., 2017). These two potent inflammatory substances damage endothelial cells and induce the expression of adhesion factors through oxidative stress and promote monocytes to aggregate into the lumen to form macrophages. Macrophages devour ox‐LDL and become foam cells, which can stimulate the proliferation of vascular smooth muscle cells, promote the formation of atherosclerotic plaques, induce the release of various inflammatory factors, and stimulate the up‐regulation of Lp‐PLA2 expression. Subsequently, more Lp‐PLA2 is produced in plaques and the vulnerability of plaques is increased, which promote plaque rupture and cause more Lp‐PLA2 to be released into circulation (Ye et al., 2015). It has been revealed in a study on ischemic stroke that (Wei, Ke, Zhao, & Cai, 2017) the plasma level of Lp‐PLA2 is significantly higher in the ischemic stroke group than in the control group, and the difference was statistically significant (*p* < .05) and that this was more significant in patients with atherosclerotic stroke in the great arteries. Hence, this may also be a risk factor for the early deterioration of central nervous system function (Wang, Hu, et al., 2018). A latest study revealed that (Wang, Zhou, et al., 2018) Lp‐PLA2 level may be a potential biomarker for detecting asymptomatic cerebral artery stenosis in adults. Furthermore, a foreign study reported that Lp‐PLA2 level was a new independent predictor for ischemic stroke. The present study revealed that NBP can inhibit and alleviate the inflammatory reaction induced by Lp‐PLA2.

Hypersensitive C‐reactive protein and CRP were actually the same substance (Koizumi et al., 2007). The expression of CRP in the human body is relatively low. Hence, in the present study, hs‐CRP was chosen for determination. A study revealed that (Wickramatilake, Mohideen, Withanawasam, & Pathirana, 2015) hs‐CRP can bind to lipoproteins and activate the classical complement activation pathway and that activating the complement component can directly activate resting vascular endothelial cells, thereby mediating the rolling, adsorption and exudation of leukocytes in endothelial cells, and the formation of unstable plaques. Infection factors further promote local immunity in unstable plaques and aggravate the apoptosis of vascular endothelial cells, plaque ulcers, and thrombosis, causing ischemic stroke. There are hs‐CRP receptors in monocytes and granulocytes, and hs‐CRP is activated by its receptor, which causes cell damage through direct infiltration and aggregation, or indirect action (cytokine production). Furthermore, hs‐CRP itself can promote the release of tissue factors from monocytes, inducing abnormal platelet aggregation and release. This thereby affects the process of coagulation and hemostasis, leading to the imbalance of the coagulation mechanism and increased risk of cerebrovascular accidents. Domestic and foreign scholars have revealed that hs‐CRP is not only a marker of vasculitis (Li et al., 2016), but also a proinflammatory factor correlated to the occurrence, evolution, and progression of atherosclerosis. Furthermore, it was also revealed that hs‐CRP is correlated to the severity, size of the lesion, and neurological deficits of cerebral infarction and that hs‐CRP can also be used as a prognostic indicator of cerebral infarction (Wang, Jiang, Du, Zhang, & Wang, 2014). The present study revealed that NBP could reduce the inflammatory effect of hs‐CRP.

Dl‐3‐n‐butylphthalide is a drug developed independently for the treatment of ischemic stroke in China, which is an effective component obtained from celery seeds (Tian, Wang, Wang, Zhang, & Zhou, 2017; Xiong et al., 2017), and it can be artificially synthesized. Some Chinese scholars (Wang, Ma, et al., 2018) have summarized the research progress of NBP in the treatment of ischemic stroke in the past 20 years, and it was considered as a promising drug for ischemic stroke. A large number of experiments at home and abroad have revealed that NBP can regulate brain energy metabolism (Yan, Wang, Yao, Liu, & Xiao, 2017), protect the mitochondria (Min et al., 2014), promote normal blood flow in the ischemic area (Xu et al., 2017), inhibit oxidative stress and platelet aggregation (Li et al., 2018; Zhao, Li, Zhang, Chen, & Li, 2013), and prevent the vigorous apoptosis of cells (Min et al., 2014). In addition, Chinese scholars found that NBP plays a neuroprotective role by acting on multiple active targets (Wang et al., 2017; Zhao et al., 2017). Furthermore, some scholars considered that the effective rate of NBP in treating ACI can reach 76%–93% (Offner et al., 2006). This is consistent with that in the present study.

The results of the present study revealed that NBP can reduce the levels of Lp‐PLA2 and hs‐CRP in patients with ACI. It also confirms the anti‐inflammatory, antioxidant, and antithrombotic effects of NBP. Furthermore, it was revealed that although both Lp‐PLA2 and hs‐CRP are independent predictors of acute stroke, the combined analysis of these two can better predict the progression and evolution of stroke. Some scholars (Liu et al., 2018) have performed some work in this field.

## 5 CONCLUSION

The present study revealed that in ACI patients treated with NBP, severe neurological deficits were alleviated, the expression of Lp‐PLA2 and hs‐CRP in blood was downregulated, and the changes in Lp‐PLA2 and hs‐CRP levels were closely correlated to neurological deficits. NBP alleviates inflammatory response by lowering the levels of Lp‐PLA2 and hs‐CRP, thereby helping patients recover neurological function. The combined detection of Lp‐PLA2 and hs‐CRP can better determine the course and prognosis of cerebral infarction.

## CONFLICT OF INTEREST

None of the authors have any financial disclosure or conflict of interest.

## Data Availability

The data that support the findings of this study are available from the corresponding author upon reasonable request.
